# Beyond mean corpuscular volume: the role of neutrophil and monocyte volume cell population data in the diagnosis of vitamin B12 deficiency in anemic patients

**DOI:** 10.1515/almed-2025-0097

**Published:** 2025-08-22

**Authors:** Alba Leis-Sestayo, Álvaro Piedra-Aguilera, Laura Jiménez-Añón, Jennifer Rodríguez-Domínguez, Carme García-Martín, Alicia Martínez-Iribarren, Cristian Morales-Indiano

**Affiliations:** Clinical Analysis and Biochemistry Department, Germans Trias i Pujol University Hospital, Badalona, Barcelona, Spain

**Keywords:** vitamin B12, cell population data, mean corpuscular volume

To the Editor,

Vitamin B12 (B12) deficiency is the primary cause of megaloblastic anemia [1]. It may result from dietary insufficiency, malabsorption due to gastrointestinal disorders or inherited defects affecting cobalamin transport or absorption. Clinically, vitamin B12 deficiency can result in a broad spectrum of hematologic and neurologic manifestations. Early and accurate diagnosis is crucial to prevent irreversible neurological damage.

The most common hematological features of B12 deficiency include anemia, macrocytosis, hypersegmented neutrophils and megaloblastic changes in peripheral blood and bone marrow, all of which reflect impaired DNA synthesis [1], 2]. However, the presence of hypersegmented neutrophils or elevated mean corpuscular volume (MCV) in peripheral blood smears are not sensitive indicators of B12 deficiency [1], 3], 4].

The Beckman Coulter DxH 900 hematology analyzer provides cell population data (CPD) using VCS technology, which integrates volume (V, via impedance), conductivity (C, via radiofrequency opacity), and light scatter (S, via multi-angle laser light scatter) to assess cellular morphology and internal complexity. This enables the generation of quantitative data on white blood cells morphological and functional characteristics.

We evaluated and compared the diagnostic performance of MCV and CPD in identifying B12 deficiency in primary care patients. This retrospective study was conducted in Spain from January 2022 to March 2024. Patients were classified into three groups: the control group (B12>300 pg/mL), group 2 (patients with B12 deficiency, B12<187 pg/mL), and group 3 (patients with severe B12 deficiency, B12<100 pg/mL). The control group was divided into two subgroups, 1a and 1b, to balance the sex distribution.

All patients presented with anemia (defined as hemoglobin <12 g/dL for women and <13 g/dL for men), ferritin levels between 30 and 150  ng/mL, white blood cells count <10 × 10^9^/L, and alanine aminotransferase <50 IU/L. Exclusion criteria included: estimated glomerular filtration rate <40 mL/min/1.73 m^2^, C-reactive protein >10 mg/L, diagnosed thalassemia, or pregnancy. Age range was 18–80 years. Patients with folate deficiency were excluded from the control groups. The Ethics Committee reviewed and approved the study (CEIC code PI-24-220).

Statistical analysis was performed using MedCalc v19.6 (Ostend, Belgium). Student’s *t*-test and receiver operating characteristic (ROC) curves were used to assess the diagnostic utility of MCV and CPD.

**Figure 1:**
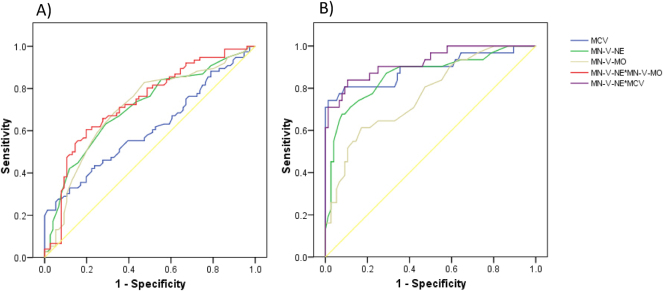
ROC curves comparing controls and patients with B12 deficiency: (A) <187 pg/mL, (B) <100 pg/mL.

We collected data on MCV and CPD parameters, including volume, conductivity and light scatter. Among these, the mean neutrophil volume (MN-V-Ne) and mean monocyte volume (MN-V-Mo) showed significant differences between control and B12-deficient groups (Table 1). In the comparison between group 1a and group 2, both MN-V-Ne and MN-V-Mo outperformed MCV in diagnostic accuracy (AUC =0.709 and 0.706, respectively). When combined (MN-V-Ne × MN-V-Mo), diagnostic performance improved further (AUC=0.732; sensitivity =60.5 % and specificity =80.2 %, cut-off >269.4) (Table 1A, Figure 1A). In the comparison between group 1b and group 3, both MCV and MN-V-Ne showed strong diagnostic performance, and their combination (MCV × MN-V-Ne) yielded the highest accuracy (AUC=0.921; sensitivity=83.9 %, specificity=89.8 %, cut-off >141.5) (Table 1B, Figure 1B).

**Table 1:** Comparative analysis of cell population data between controls and patients with B12 deficiency: (A) B12<187 pg/mL and (B) B12<100 pg/mL.

(A)					
	Group 1a (mean ± SD)	Group 2 (mean ± SD)	p-Value	AUC (Confidence interval 95 %)	p-Value
Male/female (total)	36/40 (76)	36/40 (76)			
Age, years	64.6	65.6			
Vitamin B12, pg/mL	467.8 ± 145.4	145.1 ± 31.0			
Hemoglobin, g/dL	11.9 ± 0.8	11.7 ± 0.8			
MCV, fL	88.7 ± 5.1	92.8 ± 9.7	0.0015	0.608 (0.525–0.686)	0.019
MN-V-Ne	148.1 ± 6.2	152.8 ± 7.6	<0.001	0.709 (0.630–0.780)	<0.001
MN-V-Mo	174.6 ± 7.8	179.8 ± 8.6	<0.001	0.706 (0.627–0.777)	<0.001
MN-V-Ne x MN-V-Mo	258.8 ± 20.0	275.2 ± 24.8	<0.001	0.732 (0.654–0.801)	<0.001

(B)					
	Group 1b (mean±SD)	Group 3 (mean±SD)	p-Value	AUC (Confidence interval 95 %)	p-Value
Male/female (total)	19/40 (59)	10/21 (31)			
Age, years	64.1	68.6			
Vitamin B12, pg/mL	465.3 ± 144.1	86.7 ± 5.6			
Hemoglobin, g/dL	11.8 ± 0.7	11.3 ± 1.0			
MCV, fL	89.2 ± 4.7	105.4 ± 12.3	<0.001	0.887 (0.803–0.944)	<0.001
MN-V-Ne	148.0 ± 6.5	161.0 ± 10.8	<0.001	0.864 (0.775–0.927)	<0.001
MN-V-Mo	174.3 ± 8.3	184.9 ± 11.8	<0.001	0.776 (0.676–0.857)	<0.001
MCV x MN-V-Ne	132.0 ± 8.5	170.7 ± 29.8	<0.001	0.921 (0.845–0.967)	<0.001

SD, standard deviation; AUC, area under the curve; MN-V-Ne, mean neutrophil volume; MN-V-Mo, mean monocyte volume; p, p-value. MN-V-Ne × MN-V-Mo and MCV × MN-V-Mo represent the product of both parameters, divided by 100.

Saha et al. [5] and Simón-López et al. [6] previously reported associations between neutrophil and monocyte volumes and low B12 or folate levels, using the older Beckman Coulter LH 750 analyzer. In our study, we confirmed significant differences in MN-V-Ne and MN-V-Mo between control and B12-deficient groups using the newer DxH 900 analyzer. Furthermore, we demonstrated that the severity of B12 deficiency substantially impacts the diagnostic performance of MCV and CPD.

In the context of B12 deficiency, MCV has limited diagnostic sensitivity, particularly in mild cases. However, in severe B12 deficiency (B12 <100 pg/mL), the diagnostic performance of MCV improves significantly, making it a valuable tool for identifying B12 deficiency.

The diagnostic performance of mean corpuscular volume (MCV) can be further enhanced by incorporating cell population data (CPD). The integration of estimated reference intervals [7], similar to how MCV is used, or potential diagnostic cut-off values for these CPD parameters into laboratory information systems, may improve the identification of vitamin B12 deficiency.

One limitation of the study is the lack of evaluation of the impact of concomitant folate and B12 deficiencies. Folate deficiency is also associated with megaloblastic changes in hematopoietic cells, and the coexistence of both deficiencies could enhance their effect on diagnostic performance.

In conclusion, CPD parameters are valuable tools for detecting B12 deficiency in anemic patients. These markers complement MCV and provide additional diagnostic support at no extra cost, as they are automatically generated during routine complete blood count. This approach could enable more precise and timely diagnoses, particularly in complex cases of B12 deficiency or when MCV is altered due to other types of anemia, treatments, or underlying conditions.
